# Active sampling of volatile chemicals for non-invasive classification of chicken eggs by sex early in incubation

**DOI:** 10.1371/journal.pone.0285726

**Published:** 2023-05-22

**Authors:** Eva Borras, Ying Wang, Priyanka Shah, Kevin Bellido, Katherine L. Hamera, Robert A. Arlen, Mitchell M. McCartney, Kristy Portillo, Huaijun Zhou, Cristina E. Davis, Thomas H. Turpen

**Affiliations:** 1 Mechanical and Aerospace Engineering, University of California Davis, Davis, CA, United States of America; 2 UC Davis Lung Center, Davis, CA, United States of America; 3 Department of Animal Science, University of California Davis, Davis, California, United States of America; 4 SensIT Ventures, Inc., Davis, CA, United States of America; 5 VA Northern California Health Care System, Mather, CA, United States of America; Accra Technical University, GHANA

## Abstract

According to industry estimates, approximately 7 billion day-old male chicks are disposed of annually worldwide because they are not of use to the layer industry. A practical process to identify the sex of the egg early in incubation without penetrating the egg would improve animal welfare, reduce food waste and mitigate environmental impact. We implemented a moderate vacuum pressure system through commercial egg-handling suction cups to collect volatile organic compounds (VOCs). Three separate experiments were set up to determine optimal conditions to collect eggs VOCs to discriminate male from female embryos. Optimal extraction time (2 min), storage conditions (short period of incubation during egg storage (SPIDES) at days 8–10 of incubation), and sampling temperature (37.5°C) were determined. Our VOC-based method could correctly differentiate male from female embryos with more than 80% accuracy. These specifications are compatible with the design of specialized automation equipment capable of high-throughput, *in-ovo* sexing based on chemical sensor microchips.

## Introduction

It is widely appreciated that a variety of new technologies and human behavioral change will be required to feed an estimated population of 10 billion people by 2050 with environmentally sustainable production. It is also estimated that about one third (in weight) of the world’s food is lost or wasted annually. In 2021 the poultry industry supplied some 286 eggs per capita to consumers in the US market alone [1]. Remarkably, this has been achieved with an enormous reduction in the environmental impact per kilogram of eggs produced in the past 50 years [2]. Most of the production efficiencies can be attributed to the improved performance of modern laying breeds. However, the cockerels of laying hens are generally not used for meat production. Consequently, it is estimated that some 7 billion day-old male chicks are annually culled worldwide [3]. If the sex of the eggs could be determined early in incubation, hatcheries would be able to more humanely divert male eggs for other use thus improving both the economics of production and reducing waste [4]. There has been important initial commercial success with *in ovo* sexing by automating detection of sex hormones, metabolites and DNA markers, but these methods require sampling allantoic fluid or embryo-derived cells and are not readily amendable to multiplexing [5].

In response to consumer demand, the industry is seeking a high-throughput, process engineering solution to identify the sex of a fertilized developing embryo without disrupting the egg shell and membrane surfaces. Ideally, this method would be implemented without significantly increasing food costs. Technically, this is essentially an information problem. Information can non-invasively be transmitted through such a structure in two fundamental ways, as photons or trace gases. Optical methods alone have thus far not provided the high level of specificity and sensitivity necessary to accurately classify the sex of the embryo in a scalable, practical process [6]. The egg shell is a complex highly porous structure, evolved to provide homeostatic gas exchange, physical integrity, and hatching success. Early publications demonstrated that biological information is encoded in volatile organic compounds (VOCs) that are emitted through the egg shell from avian species including chickens [7–9].

A more recent publication [10] describes "sex-specific" VOCs collected from the eggs of commercial chicken varieties by exposing solid-phase microextraction fibers (SPME) in an enclosed headspace system for one hour using eggs sealed in custom glass containers [11]. In the present study, we use an active sorptive sampling system that more rapidly collects egg VOCs with as little as 2 min of sampling time. Afterwards, samples were analyzed by thermal desorption-gas chromatography-mass spectrometry (TD-GC-MS). Data confirmed the presence of many up and down regulated VOCs that can be statistically analyzed to discriminate the sex of the embryo with low collection times. Because the method is open, operates with the commercial vacuum cups and pressures currently used to handle eggs in existing automation equipment, and is compatible with novel multiplexing sensor hardware, this approach warrants further investigation to optimize sampling conditions.

## Materials and methods

### Egg incubation and SPIDES treatment

Eggs from the Hy-Line commercial layer variety W-36 were used in this study. Eggs were either stored at cold temperatures of 13–14°C or incubated at temperatures of 37.4–37.5°C with relative humidity around 50–60%. Experiment A was first performed using only fresh eggs. Since a short period of incubation during egg storage (SPIDES) improves the hatchability of eggs stored longer than 8 days [12] some eggs were moved to the incubator for a period of several hours before returning to cold storage. For experiment B two SPIDES treatments were used after 5 and 10 days of cold storage before the start of incubation on day 15 of cold storage. For experiment C only one SPIDES treatment was performed after 6 days of cold storage starting the incubation on day 12 of cold storage. Fresh eggs in experiment A and B were in cold storage for less than 2 days.

In this study eggs were incubated a laboratory hatcher (Brinsea Products, MJ1023C OVA-Easy Advance Series II Hatcher) with a capacity of 96 hen eggs.

### Molecular sexing

High quality genomic DNA was extracted from embryo blood and allantoic fluids by using the DNeasy Blood and Tissue kit (Qiagen, Germantown, MD). In brief, embryo blood and allantoic fluid samples were collected from different developmental stages and batches. Then 100ul of each sample was incubated at 56°C with 20ul proteinase K and 180ul ATL buffer overnight (or until homogeneous). After the incubation, 200ul ethanol (96–100%) was added to each sample, mixing thoroughly, and transferred to the DNeasy Mini spin column. The manufacturer’s protocol was followed for DNA binding, washing, and elution. DNA concentration was measured by Nanodrop 2000c (Thermofisher, Waltham, MA). DNA integrity was checked by gel electrophoresis. High quality genomic DNA samples were stored at -20°C for later usage. For the determination of embryo sex, two pairs of primers were used to amplify one specific fragment on the W chromosome and another specific fragment on the Z chromosome, respectively. The primer sequence pair USP1/USP3 was used to amplify the W chromosome and CPE15F/CPE15R was used to amplify the Z chromosome [13]. PCR was carried out in a 25ul reaction system containing 0.2nM of dNTP, 0.4μM of primers, and 3 units of Taq polymerase (NEB, Ipswich, MA). The PCR conditions were 95°C 5 min, followed by 35 cycles at 95°C 80s, 60°C 90s, 72°C 60s, and a final extension at 72°C for 5 min. PCR products were loaded on 1.2% agarose gel and run in a Tris-acetate-EDTA buffer at 100 V for 25 min. All PCR products should have amplicons for the CPE15F/CEP15R primer set since both male and female embryos carry the Z chromosome. Only female embryos have amplicons for the USP1/USP3 primer set because only females carry the W chromosome. Sex information of all samples were recorded on Excel spreadsheets.

### Candling and euthanasia

Fertile eggs were distinguished from unfertilized eggs or embryos that fail to develop by candling on day 7 of incubation. After the eggs were sampled, chicken embryos were euthanized by CO2 treatment under a local IACUC-approved protocol (SIET19, 9/11/19) [14].

### Egg VOC collection

Silicone suction cups commonly used for commercial egg handling (VSO 33 SIT5, Coval) were placed individually over each egg and contained three sterile and pre-conditioned HSSE (Headspace Sorptive Extraction) magnetic stir bars (’Twisters^®^’, Part 011222-001-00, Gerstel US, Linthicum Heights, MD), providing three technical replicates per sample. HSSE commercial bars measured 10 mm length and were coated with 0.5 mm thick layer of polydimethylsulfide (PDMS) sorbent. Three Twisters^®^, which provided three technical replicates, were held in place to the inside top wall of the suction cup with a small neodymium magnet placed on the outside of the suction cup. Twisters^®^ were exposed to the top of the eggs over the air sac for between 30 s and 10 min to collect the VOCs, after which the cup was removed from the eggs. Vacuum flow was applied to move the air through the Twisters^®^ during the exposure time using a micropump device [15] used previously for environmental sampling [16, 17]. The flow was approximately 50 ml per min, measured in this experimental set-up with a mass flow controller. Air blanks were included from the adjacent laboratory or surrounding incubator air of the eggs that were sampled in each experiment in this open system.

Simultaneously, for a few of the samples, a proprietary micro-preconcentrator chip (μPC) [18] was placed downstream of the Twisters^®^ for additional VOC collection by an alternative VOC collection technology and sorbent type. While described elsewhere, briefly, the μPC chips are fabricated into glass substrates using lithography followed by etching flow channels and the cavity for the sorbent. Heaters and resistance temperature detectors (RTDs) were added to the backside of the bonded μPC to achieve rapid heating of the sorbent cavity to desorb the VOCs for detection. They are packed with Tenax TA sorbent [18].

### Experimental design

Our preliminary experiments based on sealed headspace sampling failed to yield satisfactory results. We were also concerned about possible unanticipated and unknown effects of gas exchange with decreasing oxygen and increasing carbon dioxide concentrations over time in a sealed system. Likewise, it is difficult to control for temperature and humidity. With a living system, it seems possible that this lack of environmental control might introduce metabolic perturbations that manifest as additional variation in VOC concentrations. Therefore, three separate experiments were set up to determine which conditions were more adequate to collect eggs VOCs for sex discrimination (Table 1) by active sampling. First experiment (A) was set up to define the collection flow conditions using 12 fresh eggs after 10 days of incubation. All eggs were stored at incubator temperature (37.5°C) and Twisters^®^ were exposed for 5 min. A larger experiment (B) was subsequently applied using 36 eggs applying flow with 2, 5 and 10 min of Twister^®^ exposure time. Different incubation days were studied (8–10) with fresh and SPIDES eggs. Finally, a similar experiment (C) was defined to check shorter exposure times (30 sec) and incubation temperatures. For that, 36 eggs were used, all fresh and around day 8 of incubation, where 12 were exposed for 30 sec at room temperature, and 24 were exposed for 2 min. From those, 12 were stored in the incubator and 12 at room temperature.

**Table 1:** Experiments tested to define best experimental conditions to collect sex discriminatory VOC from eggs.

Experiment	Egg type	VOC extraction time	Incubation day	Temperature incubation	Flow	Total egg number
A	Fresh	5 min	Day 10*	Incubator at 37.5°C	Flow No flow	12
B	Fresh SPIDES	2 min 5 min 10 min	Day 8 Day 9 Day 10	Incubator at 37.5°C	Flow	36
C	SPIDES	2 min 30 sec	Day 8*	Room at 21°C Incubator at 37.5°C	Flow	36

* During these 2 experiments, there were intermittent overnight power outages to the incubator for unknown periods of time. Reduction in degree days may have reduced the physiological age of the eggs.

### Twister^®^-GC-MS analysis

We used around 12 egg replicates per each condition and three technical replicates (Twister^®^ sorbent bars) per each egg. All Twisters^®^ were pre-conditioned prior to use, according to manufacturer specifications, and blank air samples from each condition was simultaneously collected. All Twisters^®^ were placed into 2 ml borosilicate vials and stored at -20°C before the analysis.

Before the analysis, each Twister^®^ was transferred into thermal desorption tubes and 1 *μ*l of internal standard (500 ppb naphthalene-D8 in ethanol solution) was added. Then, Twisters^®^ were thermally desorbed using a thermal desorption unit (TDU, Gerstel US) and cooled injection system (CIS, Gerstel US). The TDU was initially set to 50°C for 0.2 min and heated at 60°C min^−1^ until reaching 250°C and held for 4 min. A flow of helium led desorbed analytes into the CIS, which was held at −80°C. After desorption, the CIS heated at 12°C s^−1^ to 260°C and was held for 3 min. This process injected analytes in a splitless mode onto the head of the GC column.

An Agilent 7890A GC (Agilent Technologies Inc., Santa Clara, CA) equipped with a DB-5ms column (30 m **×** 250 *μ*m **×** 0.25 *μ*m, Agilent Technologies Inc.) was used for the separation of analytes. The column was initially held at 40°C for 3 min, then heated at 3°C min^−1^ to 150°C. After the oven reached 180°C using 10°C min^−1^, and then was heated at 30°C min^−1^ to 300°C and held for 7 min. Total runtime was 53.66 min. The GC worked in constant flow mode at 1.5 ml min^−1^ of helium. Analytes eluted into a 5975C single quadrupole mass spectrometer (MS, Agilent Technologies Inc.). The MS scanned from 40 to 300 *m*/*z*. Its source and quad were set to 230°C and 150°C, respectively.

A bake out of the TDU–CIS–GC–MS system was conducted every ∼20 injections. After every 30–40 GC–MS injections, a standard mixture of C_8_-C_24_ alkanes was analyzed to serve as an external control of the instrument and to calculate Kovats retention indices of compounds.

Similar to Twister-GC-MS analysis, μPCs were loaded onto a custom aluminum test fixture connected to the injector of a GC-MS for chemical analysis. In brief, the chip was heated and held at ∼260°C for 15 min under a 25 mL min−1 flow of helium. A borosilicate transfer line connected desorbed analytes to the GC-MS inlet, and VOCs were analyzed using the GC-MS method as described above. All compounds were eluted within 26.6 min.

### GC-MS data analysis

Raw data was initially checked with Agilent’s Mass Hunter Qualitative Analysis B.06.00 software for qualitative reasons. Deconvolution and alignment of the GC–MS data files was achieved using recursive feature extraction on Profinder (Version B.08.00, Agilent Technologies Inc.) and Mass Profiler Professional (MPP, V13.0). This process provided a peak table with samples in columns and variables or features in rows, containing peak areas or intensities.

Features corresponding to siloxane base peaks (207, 221 and 281 *m*/*z*) were initially removed. Then, the peak table was cleaned by removing features that were missing in more than 20% of samples and appear in blank samples with signals higher than 3 (peak sample/blank ratio). Three different blanks were used to remove features not specific from the samples: system blank (using an empty thermal desorption tube), Twister® blanks (clean twisters), and air blanks (collected in the environment of the experiments). All missing values were replaced by minimum positive value divided by 10. Heteroscedasticity was corrected to the final datasets using log transformations.

Statistical analyses were performed using Excel, MATLAB R2017a and PLS Toolbox (Version 8.6, Eigenvector Research Inc., Manson, WA) software. Comparative statistics of means to assess the significance of the changes using t-tests/Wilcoxon rank sum test. A *p*-value of *p* < 0.05 was used throughout for significance. An initial variable reduction was performed based on statistical significance (p < 0.05). Multivariate models were used for comparative analyses: principal component analysis (PCA) and Partial Least-Squares Discriminant Analysis (PLS-DA). PCA was performed initially to visualize similarities between observations and detect potential outliers in an unsupervised way. PCA projects the maximum variance of the dataset in a linear additive model. Principal components (PC) are orthogonal variables that rank variances of features and reduce the dimensionality of the multivariate data set [19]. Finally, PLS-DA was used as a classification method, with a supervised approach, it uses correlation between the dataset of features and a matrix of known responses that contains sample information and classes/groups [20]. PLS-DA separates different groups of samples based on their features. Classification ability is determined by sensitivity (probability to correctly detect a class), specificity (ability to correctly reject a class) and area under the curve (AUC) values, which are defined by receiver operating characteristic (ROC) curves [21]. AUC measures the classification ability at different thresholds, telling how much a model is able to distinguish classes or groups. Means and standard deviations of classification abilities were defined by cross-validation and prediction sets. For that, the data was randomly split 50 times using 67% for samples for a calibration training set and 33% for a prediction set (not included to build the model). Cross-validation was performed using the venetian blinds technique, where the calibration data were split into 10 random splits and one sample per split was used to cross-validate the model.

PLS-DA also provides a list of potential markers related to a defined class ranked by variable importance in projection (VIP) values. VIPs summarize the impact of each feature to the model and values higher than 1 are considered relevant for that classification [22]. This allowed an additional variable reduction using VIP values (VIP > 1). Identifications of these relevant features or potential markers were performed later by searching through a commercial database (NIST 20) along with comparison of calculated Kovats Retention Index values to the ones reported in literature. Identifications were tentatively described when experimental and theoretical spectral pattern (scores) of the compounds were higher than 65–70%.

## Results and discussion

Initial raw dataset contained 568 features. After data processing and filtering, the number of features was reduced to 222 and each experiment was individually studied.

### Experiment A: Active versus passive VOC extraction

The optimization of starting conditions was focused on the active or passive sampling of the VOCs (Table 1, Experiment A). Twisters^®^ were exposed with and without active flow to capture the VOCs from the eggs. In this case 12 fresh eggs were used after 10 days of incubation at 37°C. Each Twister^®^ was exposed for 5 min, using 15 min of exposure for some of the “non-flow” samples. From the resulting eggs, PCR results and candling showed that 3 were non-viable or unfertile, 5 male and 4 female.

**Fig 1:**
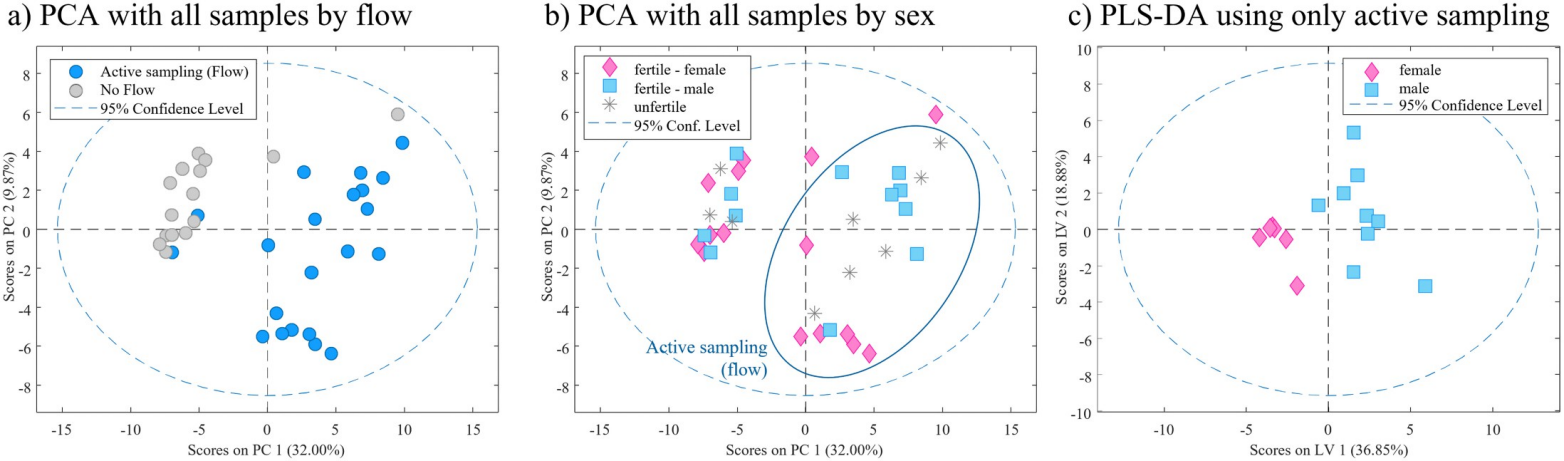
Results of the use of active sampling in VOCs detection in Experiment A. (a and b) PCA scores plot showing differences between active and non-active sampling: (a) and distribution of samples by sex (b). (c) PLS-DA scores plot applied to only active sampled eggs (using flow) shows initial differences between sex of the samples.

Initial data visualization showed clear differences between samples exposed using the active sampling (flow) and non-flow (Fig 1A). When samples were presented by sex defined by PCR technique (Fig 1B), we observed some sex related differences within only the samples corresponding to the active sampling. In this case, unfertile samples were also plotted, but no relevant “sex” information was provided from those samples.

Finally, PLS-DA was applied to just fertile samples using active sampling (Fig 1C), obtaining high AUC values (0.92 ±12.2%). High variability (>25%) was also achieved for sensitivity (0.91) and specificity (0.77) values, mainly caused by the low number of samples represented in each group (2 female and 3 male). However, we could determine that around 10 days of incubation and the use of active sampling was sufficient to show a trend to the sex differentiation.

### Experiment B: Egg storage conditions

**Fig 2:**
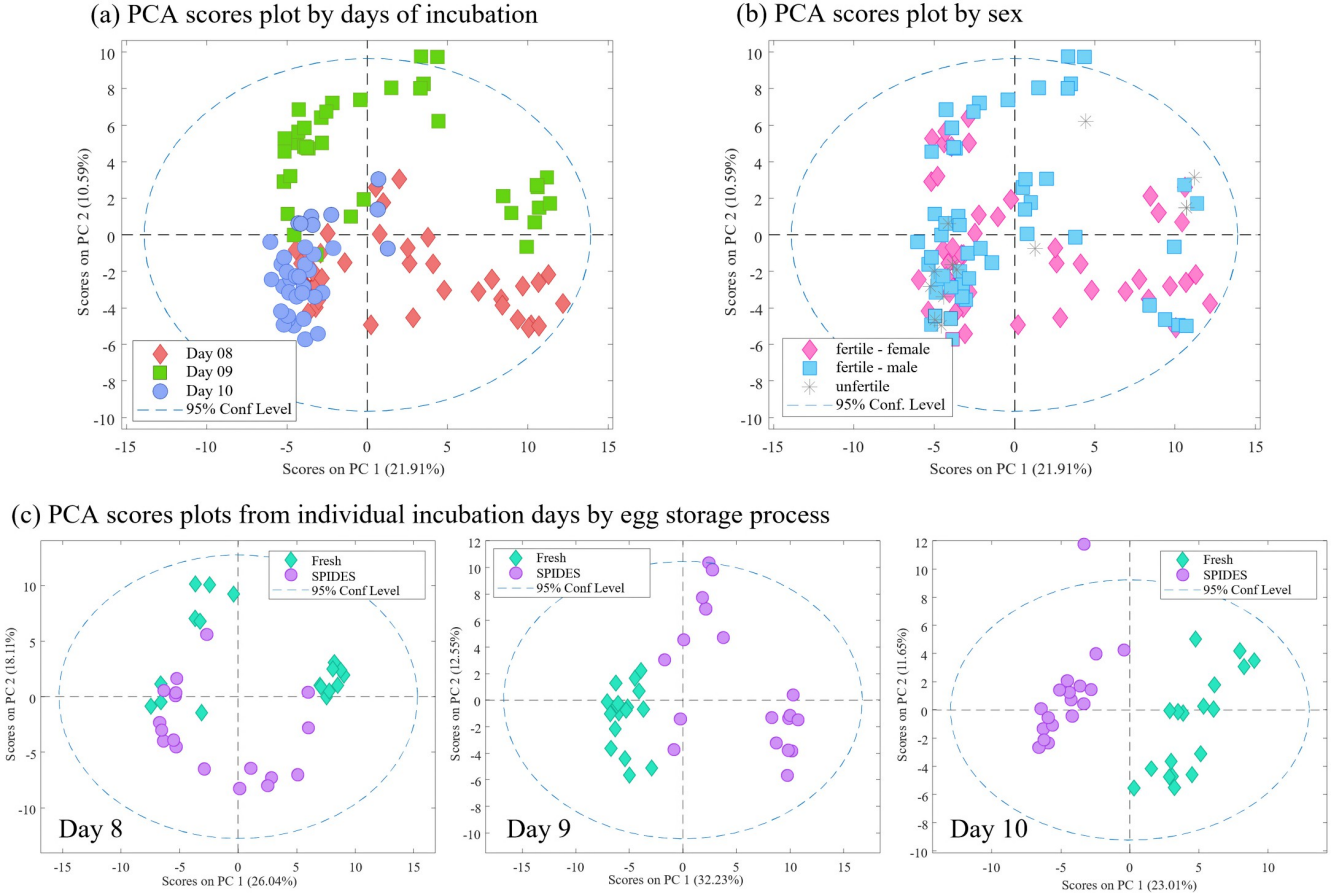
Results of Experiment B for VOCs detection. (a and b) PCA scores plot showing differences between days of incubation (a) and sex (b). (c) PCA sores plots between egg storage process (fresh vs. SPIDES) within the different days of incubation.

Once active sampling was defined as a requisite to collect VOCs, the next experiment was focused on the use of different egg storage conditions (fresh vs. SPIDES), days of incubation and time of Twisters^®^ exposure (Table 1, Experiment B). For that, 36 eggs were used: 18 fresh and 18 SPIDES, and batches of 6 eggs were exposed Twisters^®^ for 2, 5 and 10 min, using 8, 9 and 10 days of incubation. From the PCR and candling results, we found 4 unfertile eggs, 17 male and 15 female (Fig 2).

When all the samples were presented (Fig 2) main differences were shown due to the days of incubation, with some overlapping, mainly from days 9 and 10 (Fig 2A). However, when the samples were presented by sex (Fig 2B, including unfertile) no clear distribution was observed. If data was then separated by days of incubation, we could observe that main differences were due to the egg storage process, being more evident through the days (high separation in day 10, Fig 2C).

Since unfertile samples do not provide relevant information for the purpose of the study, we proceeded with only fertile samples. When PLS-DA was built with all the samples, low classification abilities were obtained (0.71 AUC and 0.62 and 0.73 for sensitivity and specificity, respectively). With that, classification models to discriminate egg sex were built using selected subgroups of samples from specific studied conditions (Table 2).

**Table 2:** Classification abilities for the PLS-DA models using different conditions.

	AUC	Sens (Pred)	Spec (Pred)
All fertile	0.71 (11.0%)	0.62 (22.2%)	0.73 (14.2%)
2 min	0.99 (2.9%)	0.89 (10.4%)	0.98 (5.8%)
5 min	0.96 (5.7%)	0.90 (7.3%)	0.88 (9.7%)
10 min	0.92 (8.6%)	0.82 (10.3%)	0.86 (11.6%)
Day 08	0.77 (16.2%)	0.73 (12.7%)	0.72 (16.3%)
Day 09	0.77 (13.6%)	0.77 (10.7%)	0.68 (16.0%)
Day 10	0.76 (14.8%)	0.75 (19.1%)	0.69 (24.3%)
Fresh	0.73 (15.9%)	0.69 (12.7%)	0.69 (15.4%)
SPIDES	0.82 (13.9%)	0.67 (13.8%)	0.81 (10.1%)

**Fig 3:**
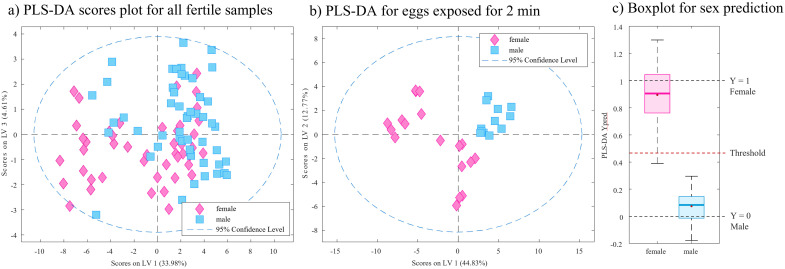
PLS-DA models by sex using fertile samples from Experiment B. (a) PLS-DA scores plot build using all fertile samples from Experiment B. (b) PLS-DA scores plot built using only eggs with Twisters® exposed during 2 min. (c) PLS-DA Y prediction boxplot showing the differences between male (Y = 0) and female (Y = 1) sample responses when applied to the model, using a Threshold value to discriminate.

From all the conditions studied, we could observe that SPIDES eggs were more accurately discriminated by sex than fresh eggs based on VOCs. Similar results were obtained for the different days of incubation, providing just slightly better classification abilities compared to all samples together (all fertile). However, we could determine that 2 min of exposure time offered the best separation between male and female eggs (0.99 AUC and 0.89 and 0.98 for sensitivity and specificity, respectively) (Fig 3). Moreover, results were worse when time of exposure was increasing, suggesting that shorter times of Twister® exposure may be advantageous for the collection of sex discriminatory VOCs. Also, higher values on sensitivity determine that is easier to differentiate male eggs from the rest, giving the higher variability shown in female samples distribution.

### Experiment C: VOC extraction times, incubation temperatures

Knowing that short periods of Twister® exposure times had higher accuracies to discriminate eggs by sex, we decided to check even lower times of exposure (30 sec). Also, the temperature of sample collection was compared at room temperature (21°C) and incubator temperature (37.5°C) (Table 1, Experiment C). From the PCR results and candling, we found 4 unfertile eggs, 19 male and 13 female.

**Fig 4:**
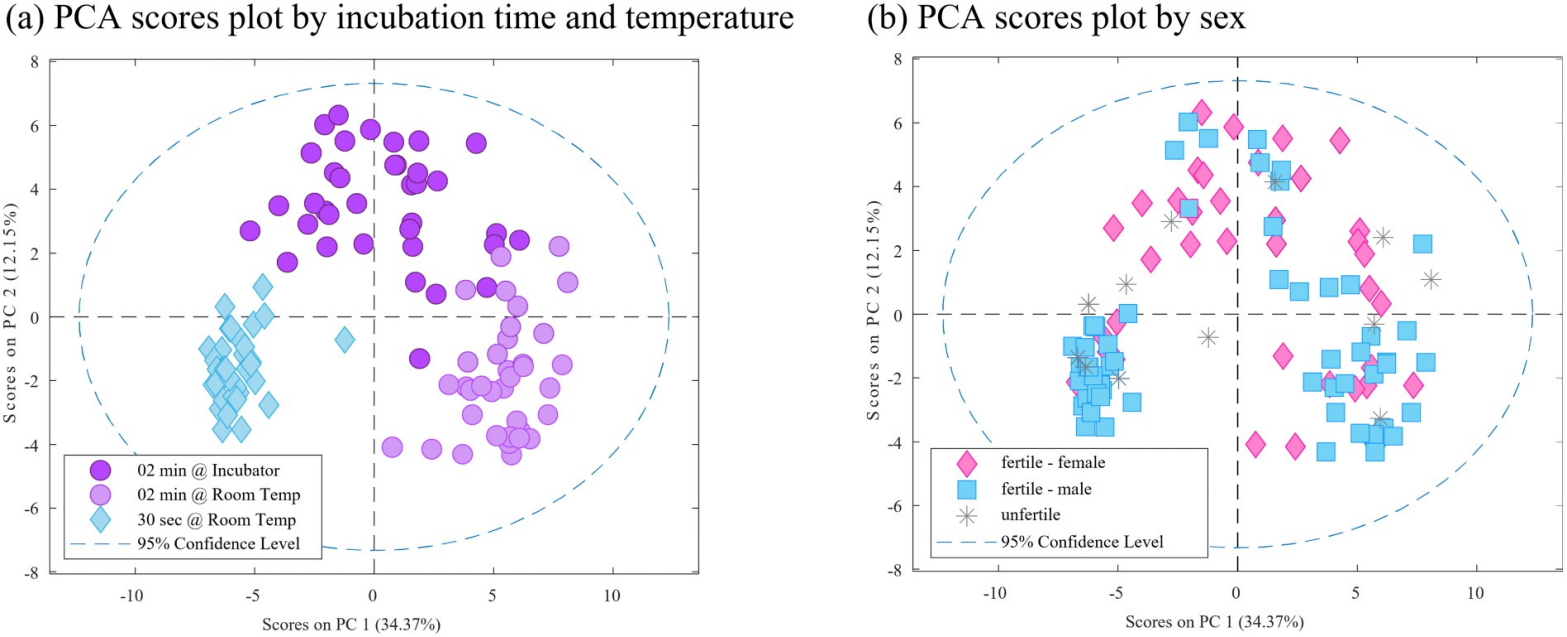
Results of Experiment C for VOCs detection. (a and b) PCA scores plot showing differences between tested conditions (a) and sex (b).

With all samples we could observe that main differences are caused by the three conditions tested (Fig 4A). And when presented by sex (Fig 4B, including unfertile) no clear distribution is observed.

After deleting unfertile samples PLS-DA showed low classification abilities (0.68 AUC and 0.63 for both, sensitivity and specificity). With that, classification models to discriminate egg sex were built using selected subgroup of samples from specific studied conditions (Table 3).

**Table 3:** Classification abilities for the PLS-DA models using different conditions.

	AUC	Sens (Pred)	Spec (Pred)
All fertile	0.68 (10.9%)	0.63 (20.8%)	0.63 (18.3%)
2 min (Lab + Inc)	0.78 (10.5%)	0.66 (24.5%)	0.76 (18.7%)
Lab (2 min + 30 sec)	0.60 (14.9%)	0.64 (24.6%)	0.53 (18.1%)
30 sec + Lab	0.48 (41.5%)	0.47 (72.6%)	0.51 (33.6%)
2 min + Lab	0.71 (19.5%)	0.68 (28.8%)	0.63 (23.9%)
2 min + Inc	0.81 (15.0%)	0.68 (24.2%)	0.75 (30.9%)

In this case, we clearly observed that shorter times, like 30 sec of exposure, using room temperature provided poor discriminations by sex. Within the 2 min of exposure, the results are clearly improved using eggs sampled at the incubator temperature instead of the ones sampled at room temperature. For this model, we detected significantly lower number of features (53 compared to 80–90 from previous experiments). It should be noted that the physiological age of the eggs during this period of incubation could be lower than 8 days in this experiment because of intermittent overnight power disruption to the incubator. This may be the reason for lower confidence in sex discrimination observed in Experiment C compared to Experiment B.

### Identification of sex discriminatory VOCs

After building discriminant models with optimal conditions, variable importance in projection (VIP) scores were generated for each feature (in this case, an egg VOC of interest). Features with VIP values greater than one are typically considered relevant to the discrimination. Initial samples collected with active sampling (Experiment A) had 25 compounds with a VIP > 1, fresh + SPIDES samples collected for 2 min between days 8–10 incubation and at 37.5 C (Experiment B) had 41 compounds, and SPIDES samples collected for 2 min at 37.5 C, but at around day 8 of incubation (experiment C) had 16 compounds. From all these compounds, only 5 were overlapped (2 between exp. A and B, and 3 between exp. B and C). The low number of overlapping compounds between experiments may be due to the multiple conditions changed that can, clearly, affect the signal detected from the VOCs.

From that, a total of 77 features were described as specific for the sex differentiation. These features were putatively identified when score values were >65% and experimental Kovats Index (KI) values were confirmed with KIs from literature. Only 40% of the VIPs were characterized, being most of them detected in experiment B. Table 4 shows these identified volatile compounds with their corresponding molecular formula, molecular weight, identification score (% score and KI), regulation, corresponding experiment, and family.

**Table 4:** Putative identifications of the 30 egg-derived compounds with the highest VIP scores for sex classification.

#	Volatile compounds	MF^a^	MW	% score	KI^b^	Reg^c^	A	B	C	Family
1	Propyl-hydrazine	C3H10N2	74	78.42	714 (715)	down			x	Hydrazine
2	1-Cyclopropylethanol	C5H10O	86	85.58	751 (780)	up			x	-
3	3-Hydroxy-2-butanone (Acetoin)	C4H8O2	88	75.17	717 (743)	up		x		Ketones
4	Styrene	C8H8	104	69.64	876 (888)	up	x	x		Benzene Derivatives
5	m/p-xylene	C8H10	106	84.3	870 (863)	up		x		Benzene Derivatives
6	Ethylbenzene	C8H10	106	84.13	885 (888)	up		x		Benzene Derivatives
7	o-Xylene	C8H10	106	76.44	910 (888)	up		x		Benzene Derivatives
8	Benzoic acid	C7H6O2	122	72.05	1180 (1178)	up		x		Benzene Derivatives
9	1-(1-Methylethoxy)-2-propanol	C6H14O2	118	74.53	792 (815)	up		x		Fatty alcohols
10	3-Methyl-2-heptanol	C8H18O	130	73.4	915 (899)	down		x		Fatty alcohols
11	2-Octanol	C8H18O	130	73.74	990 (994)	down	x			Fatty alcohols
12	2-Nonanol	C9H20O	144	76.86	1106 (1105)	down		x		Fatty alcohols
13	2,7-Dimethyl-1-octanol	C10H22O	158	72.42	1130 (1102)	up	x			Fatty alcohols
14	(E)-2-Tridecen-1-ol	C13H26O	198	66.59	1564 (1496)	down	x			Fatty alcohols
15	Isobutyl acetate	C6H12O2	116	78.19	755 (765)	up		x		Fatty acid esters
16	Methyl 2-methoxyacrylate	C5H8O3	116	67.11	729 (781)	up		x		Fatty acid esters
17	Ethyl butyrate	C6H12O2	116	67.59	805 (802)	up		x		Fatty acid esters
18	Ethyl isobutyrate	C6H12O2	116	77.07	756 (798)	down			x	Fatty acid esters
19	Isopropyl butyrate	C7H14O2	130	70.03	834 (840)	up			x	Fatty acid esters
20	Methyl hexanoate	C7H14O2	130	75.18	921 (926)	down			x	Fatty acid esters
21	Butyl butyrate	C8H16O2	144	86.71	984 (997)	up			x	Fatty acid esters
22	Ethyl hexanoate	C8H16O2	144	79.86	985 (1003)	up	x	x		Fatty acid esters
23	Hexyl acetate	C8H16O2	144	65.31	1007 (1016)	up		x		Fatty acid esters
24	1-Methyl-1-phenylethyl acetate	C11H14O2	178	70.04	1084 (1086)	down	x			Fatty acid esters
25	Carbonic acid, nonyl vinyl ester	C12H22O3	214	65.95	1414 (1371)	down		x		Fatty acid esters
26	Paraldehyde	C6H12O3	132	70.23	795 (802)	down			x	Trioxanes
27	m-Cymene	C10H14	134	73.65	1025 (1022)	up		x		Terpenes
28	D-Limonene	C10H16	136	98.1	1030 (1025)	up		x		Monoterpenoids
29	beta-Thujene	C10H16	136	73.61	971 (1007)	up		x		Polycyclic olefin
30	5,6,7,8-Tetrahydropteridine	C6H8N4	136	65.65	1424 (1403)	down	x			Pteridines
31	Decanal	C10H20O	156	71.59	1204 (1205)	up		x		Aldehyde
32	N-(3-Ethylpentyn-3-yl)pyrrolidine	C11H19N	165	70.72	1222 (1203)	up	x			Pyrrolidines
33	Tetradecane	C14H30	198	86.5	1400 (1400)	down		x		Alkanes
34	2-Myristynoyl pantetheine	C25H44N2O5S	484	71.63	4031 (>2100)	up			x	-

^a^MF: molecular formula; MW: molecular weight

^b^KI: Kovats index, calculated/experimental and (as reported in the literature)

^c^Reg: up-regulated defined as more abundant in female eggs, down-regulated defined as more abundant in male eggs

Half of the total selected features (49%) were up-regulated by female defined eggs, meaning that those compounds where characteristic by having higher intensities in the female group compared to male group.

From the compounds identified, we can observe some benzene derivatives (all up-regulated), such as xylene, styrene, ethylbenzene or benzoic acid. Several fatty alcohols were also detected, like 2-octanol, 2-nonanol, similarly to other studies where the role of sex-related pheromone alcohols have been studied [10, 11]. It’s important to note the presence of several fatty acid esters to define sex differences, such as isobutyl acetate, ethyl butyrate, isopropyl butyrate, methyl hexanoate, among others. Interestingly, one of the compounds, decanal, has also been defined as important VOC for the distinction of male and female eggs in recent studies [10, 11]. Other compounds like m-cymane, d-limonene, b-thujene or tetradecane are also listed for sex differentiation ability.

### Study with other sorbents

We used 4 uPC chips to sample 3 eggs and incubator air (blank) and identified a total of 38 compounds after 15 minutes of sampling. From these, 8 compounds were previously reported by Xiang et al. identified to be significant for *in ovo* sexing across three breeds of chickens including hexane, hexanal, heptanal, 6-methyl-5-hepten-2-one, nonanal, decanal, undecanal, and dodecanal [10].

## Conclusion

We have established an experimental method to actively sample VOCs from chicken eggs in an open system that is compatible with existing commercial vacuum-based automated egg handling equipment. Our results corroborate the recent study published by Xiang et. al. based on headspace sampling in a closed system [10]. There are abundant egg-derived VOCs that be used to statistically classify embryos by sex, non-invasively, early in incubation with high confidence. In the open system, the ability to classify male and female eggs begins to degrade sometime between 2 and 10 minutes of sampling at 50 ml/min as the air flow equilibrates with the surroundings. If necessary, a variety of sorbents may be packed into uPC chips to selectively trap compounds having the greatest discriminatory statistical power. In future work, a number of sampling variables need to be optimized including the time of sampling, flow rate and temperature. It will be important to understand if there is any contribution to the VOC profiles from the metabolic activity of the microbiome. However, we believe an approach for high throughput *in ovo* sexing based on integrating high performance chemical sensor microchips [23] with egg handling machinery in a multiplex format warrants further development.

## Supporting information

S1 Data Raw data corresponding to the three experiments presented in this paper. Excel files from Experiment A, Experiment B and Experiment C, describing samples, variables and the corresponding areas from the signals obtained using Twister-GC-MS.
(ZIP)
